# Macrophage migration inhibitory factor (MIF) in the development and progression of pulmonary arterial hypertension

**DOI:** 10.21542/gcsp.2018.14

**Published:** 2018-06-30

**Authors:** Mohamed Ahmed, Edmund Miller

**Affiliations:** 1 Neonatal-Perinatal Medicine, Pediatrics Department Cohen Children’s Hospital at New York, Northwell Health System; 2 The Center for Heart and Lung Research, The Feinstein Institute for Medical Research, Manhasset, New York, USA; 3 School of Medicine, Hofstra University, Hempstead, New York, USA; 4 The Elmezzi Graduate School of Molecular Medicine, Manhasset, New York, USA

## Abstract

Macrophage migration inhibitory factor (MIF) has been described as a pro-inflammatory cytokine and regulator of neuro-endocrine function. It plays an important upstream role in the inflammatory cascade by promoting the release of other inflammatory cytokines such as TNF-alpha and IL-6, ultimately triggering a chronic inflammatory immune response. As lungs can synthesize and release MIF, many studies have investigated the potential role of MIF as a biomarker in assessment of patients with pulmonary arterial hypertension (PAH) and using anti-MIFs as a new therapeutic modality for PAH.

## Pulmonary arterial hypertension (PAH)

**Figure 1.**
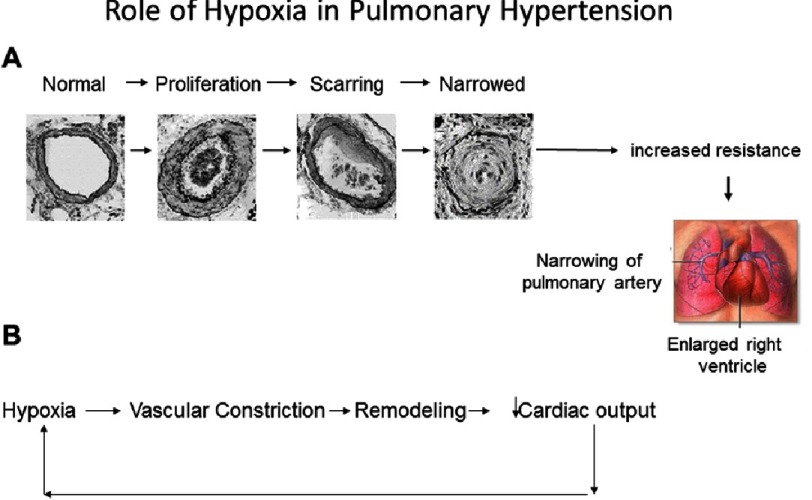
Role of hypoxia in pulmonary hypertension. (A) Hypoxia induces pulmonary vascular remodeling resulting in increased vessel wall thickness and narrowing of the vessel. The remodeling chronically increases pulmonary vascular resistance. (B) A major factor in the rapid progression of PAH pathology is due in part to the creation of a vicious cycle: Hypoxia induces vasoconstriction, which itself adds to the hypoxia, and hypoxia in return exacerbates PAH.

PAH is a devastating disease that leads to progressive systemic hypoxemia, right ventricular failure and death^1^. PAH demonstrates rapid deterioration after diagnosis, with an average survival time for primary pulmonary hypertension of only 2.8 years, and an estimated 5 year survival rate of between 21–34%^2,3^. Although previously considered a rare disease, over the last two decades, there has been an increase in the diagnosis possibly due to the increased awareness of the physician and improved diagnosis methods^4^. PAH can occur in association with chronic lung disorders, with hypoxia playing a pivotal role in the etiology. Hypoxia induces pulmonary vessel constriction and persistent hypoxia results in pulmonary vascular remodeling resulting in increased vessel wall thickness and narrowing of the vessel^5,6^ (Figure 1). Pulmonary vascular remodeling chronically increases pulmonary vascular resistance (PVR), leading to right ventricular failure, decreased left ventricular preload and reduced cardiac output. The remodeling also causes mismatch of blood flow and ventilation (V/Q), which, together with decreased cardiac output and possible cardiac shunt, lead to further hypoxia. A major factor in the rapid progression of PAH symptoms may be due in part to the creation of a vicious cycle: PAH can be initiated by hypoxia, itself causes hypoxia, and hypoxia in return exacerbates PAH.

Current therapies for PAH are relatively ineffective and their benefits limited to improving ability to exercise. They include prostacyclin and its analogues, endothelin receptor antagonists, and phosphodiesterase type 5 inhibitors^7–9^. Notably, current therapies do not significantly improve mortality, rate of clinical progression of disease, or WHO functional class^10,11^. The limitation of current treatment suggests the need for a better understanding of the pathogenesis and identification of new therapeutic targets for this lethal disease.

## Vascular remodeling

Vascular remodeling leads to increased vessel wall thickness and narrowing of the vessel lumen^5,6^. Endothelial, fibroblasts and smooth muscle (SMC) are the principal intimal, adventitial and medial cells of the vascular wall, respectively. Chronic hypoxia induces pulmonary vascular cell proliferation and remodeling, but the mechanisms involved remain unclear. While morphological changes to the intima are observed, they are usually minimal^12^. Chronic hypoxia induces structural changes to the pulmonary arteries including the appearance of SMC-like myofibroblasts expressing α-smooth muscle actin, in previously non-muscularized vessels. While hypoxia-induced remodeling is associated with medial hypertrophy, direct stimulation of SMC proliferation by hypoxia remains controversial^13^.

There are reports of hypoxia-driven smooth muscle cell proliferation^14,15^, but several *in vitro* studies have shown that hypoxia does not directly increase SMC proliferation^16–18^ or may actually decrease proliferation^19,20^. However, fibroblasts which are less differentiated than the other two cell types, have a greater proliferative response to hypoxia than either endothelial cells or SMC^20–23^. Fibroblasts are uniquely positioned in the scheme of remodeling being able to rapidly proliferate, contract, migrate, synthesize cytokines and other mediators, and transdifferentiate into other cell types such as the SMC-like myofibroblast^24^. Animal models indicate that the adventitia undergoes the earliest and most profound changes under hypoxic conditions^25,26^ and *in vitro*, hypoxia induces fibroblast proliferation in the absence of any exogenous co-mitogen^27^.

## Macrophage migration inhibitory factor (MIF)

MIF was originally described over 50 years ago in studies of delayed hypersensitivity in which it was suggested that lymphoid cells released soluble materials that inhibited the random migration of peritoneal exudate cells *in vitro*^28,29^. This resulted in the original nomenclature, but since that time, a multiplicity of functions have been assigned to the molecule has led to the less formal epithet of simply MIF^30^.

MIF is a conserved protein of 12.5 kDa, of which homologues can be found in plants, nematodes and vertebrates. In its active form, it is a homotrimer which is associated with two distinct enzymatic activities. The first, a phenylpyruvate tautomerase^31^, residing within the hydrophobic pocket between adjacent monomers. This tautomerase activity and structural relationship are similar to a second human protein, D-dopachrome tautomerase (D-DT)^32^, with which human MIF shares around 34% sequence homology. The similarities between structures and activities of the two molecules have led to the D-DT designation as ‘MIF-2’^33^.

While the relevance of the tautomerase activity to human disease remains unclear, the hydrophobic pocket within which the activity is located binds to cell surface CD74 molecules^34^ thereby activating its CD44 co-receptor initiating cellular activation, cell proliferation and inhibition of apoptosis^35,36^. The second MIF catalytic activity is a thiol-protein oxidoreductase^37^ within a conserved [Cys^57^-Ala-Leu-Cys^60^] region of the molecule. This antioxidant activity has been shown to be particularly relevant to situations of cellular oxidative stress^38–41^. Thus, these two characteristics of MIF alone make it a molecule of interest in the development and progression of pulmonary arterial hypertension.

Furthermore, MIF is a potent proinflammatory cytokine involved in both chronic and late-stage acute inflammation and plays a key role in inflammatory cell proliferation. It is stored within cytoplasmic pools and can be released to extracellular compartments following interaction with the Golgi complex-associated protein p115^42^. Extracellular release of MIF is initiated by a series of factors including cytokines, materials from Gram-positive or Gram-negative bacteria^43,44^ oxidative stress^45^, and steroids^30^. MIF is involved in wound healing^46^, synoviocyte proliferation^47^, and transformation from chronic inflammation to tumorigenesis^48,49^.

MIF increases proliferation of many cell types^48,50–54^ including fibroblasts^36,46,54–57^, endothelial cells^58^, and SMCs^59,60^. MIF also appears to be involved in systemic vascular remodeling, including carotid intima-media thickening^60,61^, and restenosis after vascular injury^60^. One possible mechanism of MIF’s proliferative effects is through the inhibition of p53, an endogenous cell cycling inhibitor that induces G1 stage arrest or apoptosis^62,63^, and is also involved in pulmonary arterial smooth muscle cell proliferation^50^.

## MIF and the lung

While early studies showed the anterior pituitary as a possible source of circulating MIF^64^, more recent studies have shown that in severe acute inflammation, the lungs are a major source of the circulating MIF, which can have a profound effect on cardiovascular function^65,66^. While MIF is a normal component in the epithelial lining fluid of the lung there is a significant increase in accumulation in the alveolar airspaces in the acute respiratory distress syndrome (ARDS)^67^. The increased lung MIF is both at the gene expression and protein levels and can be associated with haplotypes located in the 3′ end of the MIF gene^68^. Furthermore, the increased MIF concentrations due to a particular challenge, in both the alveolar spaces and the plasma, are age-dependent^69^. Thus, while increased extracellular MIF emanating from the adult lung appear to be detrimental, a recent study by Roger et al showed that in very preterm infants, low levels of MIF on postnatal day 6 were associated with an increased risk of developing bronchopulmonary dysplasia and late-onset neonatal sepsis^70^.

There are also several chronic inflammatory lung-associated pathologies that have been noted to be associated with changes in MIF. In particular, in idiopathic pulmonary fibrosis there is increased MIF expression in areas of remodeling, bronchiolar and alveolar epithelium, and ongoing fibrosis^71,72^. In systemic sclerosis, where MIF may be involved in the amplifying proinflammatory loop leading to scleroderma tissue remodeling^73^, an MIF promoter polymorphism is associated with susceptibility to pulmonary arterial hypertension in diffuse cutaneous systemic sclerosis (SSc)^74^. Functional promoter polymorphisms in the MIF gene, such as the high-expression MIF haplotype, C7, which is defined by −173*C and −794 with 7 CATT repeats, can also affect the clinical presentation of SSc^75^.

In addition, recent studies in a cohort of individuals with chronic obstructive pulmonary disease (COPD), demonstrate an association with increased plasma MIF and its acute exacerbations^76^, although others have suggested that MIF and its receptor are required for the preservation of normal alveolar structure and normal pulmonary endothelial cell apoptosis^77,78^.

## Relationship between hypoxia and MIF

Several studies describe a clear link between MIF and the presence of hypoxia. Hypoxia can lead to the secretion and elevation of MIF in fibroblasts^79^, cardiac myocyte^80^, monocytes^81^, and endothelia^81^. Hypoxia induces the stabilization of the transcription factor hypoxia inducible factor-1 alpha (HIF-1 α). When stabilized, HIF-1 α binds with aryl hydrocarbon receptor nuclear translocator (ARNT)/HIF-1 β. This hetero-dimer binds to elements found in the promoters of many hypoxia-responsive genes, leading to the expression of these target proteins such as vascular endothelial growth factor (VEGF), endothelin-1 (ET-1), and erythropoietin (EPO)^82^.

In 2006, Welford *et al*. demonstrated that the MIF gene contains an hypoxia response element within its promoter, which could explain the hypoxia induced MIF elevation^79^. This mechanism also has been suggested by others^83–86^. Furthermore, hypoxia in the presence of increased TNF α leads to an increase of the MIF receptor molecule CD44 on the surface of monocytes^87^.

Once released, MIF can contribute to hypoxic pulmonary vasoconstriction, which if maintained can lead to pulmonary vascular remodeling^88^. Zhang *et al* have shown that MIF affects delayed hypoxia-induced pulmonary hypertension and suggest that the action is via agonist enhancement on smooth muscle cells. However, while much is discussed about the apparently detrimental aspects of MIF inflammatory activity, it must be remembered that MIF has been shown to be protective in the early stages of ischemia.

Under ischemic conditions the extracellular release of MIF and its interaction with the CD74 receptor activates AMPK, thereby promoting glucose uptake and protects the cardiomyocyte^39^. In addition, the anti-oxidant activities of MIF can reduce intracellular oxidative stress and reduce injury in the post-ischemic heart^38^.

## Relationship between hypoxia, MIF, and pulmonary vascular cell proliferation

Pulmonary vascular cell proliferation is the major pathological change during hypoxia-induced remodeling. The pulmonary vascular wall is composed of three layers of different cells: endothelial cells in the intima, SMCs in the media, and fibroblasts in the adventitia. Hypoxia *in vivo* induces proliferation of all of these cells, but only fibroblast proliferation is induced by hypoxia *in vitro*^13^ in the absence of exogenous co-mitogens. In addition, fibroblast proliferation takes place earlier after hypoxic exposure than SMCs^13^, and hypoxia induces SMC proliferation only in co-culture with fibroblasts^20^.

Therefore, it appears that fibroblasts are essential to trigger the vascular remodeling process, perhaps because they are less differentiated and prepared for local injury repair^27^. Fibroblasts are remarkably plastic, allowing for rapid migration, proliferation, cytokine expression, and differentiation^27^. Fibroblasts differentiation to myofibroblasts^24^ is a critical step for vascular remodeling and hypoxia alone can induce fibroblast proliferation^20,27,89^ and differentiation to myofibroblasts^90,91^. Studies indicate that the lung is a major source of MIF^92^, is released from the lungs in patients with PAH^93^ and plays a key role in hypoxia-induced cell proliferation^45^.

## MIF and pulmonary arterial hypertension

PAH is a critical, and potentially devastating, clinical syndrome. The disorder, is particularly affects the small pulmonary arteries, and is characterized by vascular narrowing due to high-tone and abnormal vaso-reactivity. These abnormalities, if not corrected, lead to pulmonary vascular remodeling and intraluminal obstruction. Thus, the blood leaving the right side of the heart encounters an increased resistance to flow. While this can occur at any stage of life, it is particularly important in neonatal and adult pulmonary medicine.

In the neonatal setting, PH is associated with several conditions including, congenital heart disease^94^, connective tissue disease^95^ or sickle cell disease^96^, stenosis^97,98^, and chronic lung disease of prematurity^99,100^; and in adults PH is commonly seen in chronic obstructive pulmonary disease (COPD)^101–103^, sleep disordered breathing (sleep apnea)^104,105^ and sickle cell disease^106,107^.

While hypoxia plays a pivotal role in the etiology, inducing pulmonary vessel constriction, and persistent hypoxia results in pulmonary vascular remodeling, leading further narrowing of the vessel, not all individuals subjected to hypoxia or hypoxemia develop pulmonary hypertension or other sequelae.

## Inhibition of MIF activity

The importance of MIF in the pathogenesis of disease has led to the development of inhibitory strategies to try to disrupt these processes. Early studies used polyclonal antibodies to inhibit MIF inflammatory activities to prevent the lethality in rodent models of acute hepatic failure^108^ and septic peritonitis^109^.

Since that time, Phase 1 clinical trials have assessed the possible use of anti-MIF antibodies cases of malignant solid tumors, metastatic adenocarcinoma^110^, and lupus nephritis^111^. However, a disadvantage of the monoclonal antibody approach as a therapeutic pathway, is the possible development of local and systemic inflammatory reactions during administration. Therefore, a second approach whereby endogenous anti-MIF antibodies are generated, has also been advanced. This method involves active immunization with an MIF/tetanus toxin DNA vaccine and has been shown to protect against acute lung injury resulting from endotoxemia or a septic peritonitis^112^.

In 2002, Leng *et al* demonstrated that MIF interacts with the extracellular domain of the HLA class II histocompatibility antigen gamma chain (CD74), initiating activation of cell proliferation, and prostaglandin E2 production^34^. Since that time there has been considerable effort expended on developing small molecule inhibitors that could block the MIF-CD74 interaction. Studies have suggested that an imino-quinone metabolite of acetaminophen, N-acetyl-p-benzoquinone imine (NAPQI), can inhibit both the isomerase and inflammatory activities of MIF^113^.

Perhaps the most studied of these molecules is (S,R)-3-(4-hydroxyphenyl)-4,5-dihydro-5-isoxazole acetic acid methyl ester, or ‘ISO-1’^114,115^. There have been many modifications on this and other chemical scaffolds to try to develop more effective and selective inhibitory molecules^116–119^. Recently, iguratimod, a novel antirheumatic drug^120^ used in China and Japan, was found to selectively inhibit MIF inflammatory activity^121^. In many cases these molecules have been found to modify the pathology of animal models.

## Thyroxine as an MIF inhibitor

The thyroid hormone, thyroxine, 3,5,3′,5′-tetraiodothyronine (T4), has been identified as a potent inhibitor of MIF proinflammatory activities^122^. T4 is produced in the thyroid at around 100 mg/day^123^. While previously considered solely as a prohormone and parent molecule for T3 (3,3′,5-triiodo-L-thyronine), both molecules have been shown to elicit profound effects on myocardial activity^124,125^, and extra thyroidal conversion of T4 to T3, by specific deiodinases, occurs in a variety of tissues and precedes many thyroid hormone actions^126^.

Once released from the thyroid, thyroxine circulates in the blood, bound with thyroxine-binding globulin, transthyretin and albumin with only around 0.05% in the unbound, free-thyroxine (fT4) form^127^. Cellular actions of thyroxine occur at the plasma membrane, in the cytoplasm in the mitochondria or the cell nucleus^128^.

Non-genomic action of T4, which can be initiated at approximate physiological concentrations of free T4 of around 10^−10^M^129^, are initiated by interaction with the integrin *av* β3 plasma membrane receptor, or in the cytoplasm^130^. Interaction with the integrin receptor on the cell surface stimulates MAPK (ERK1/2) activation^125,129,130^, leading to a series of downstream events including nuclear-trafficking of specific proteins and serine phosphorylation of nucleoproteins^128^, including estrogen receptor-α, thyroid hormone nuclear receptor β1, and signal transducing and activator of transcription (STAT)-1 α.

**Figure 2.**
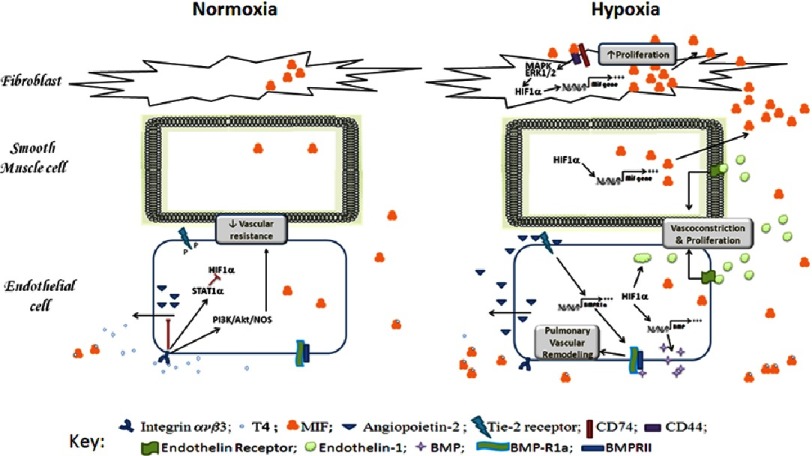
Potential mechanisms in vascular cells involving altered MIF-T4 interactions induced by hypoxia. Free thyroxine can bind to integrin avb3, decreasing extracellular ANG-2 accumulation and reducing vascular resistance. Hypoxia induces the stabilization of HIF1 *α* leading to the expression of endothelin-1 and MIF. Once released, Endothelin-1 and MIF induce cell proliferation. Hypoxia also induces production and release of BMP. Decreased fT4 (due to interaction with MIF) allows release of ANG-2 which binds its receptor TIE2 leading to transcription BMPR1a a component of the BMP receptor. Interaction between BMP and its receptor leads to altered vascular cell proliferation.

While T4, and its hormonally inactive dextro-rotary isomer D-T4, are effective inhibitors of MIF activity, triiodothyronine (T3), a T4 metabolite, is not^122^. In a clinical situation such as sepsis, plasma concentrations of T4 and MIF are inversely correlated, suggesting a clinically-relevant interaction between these two molecules^122^. In addition, a potential role of MIF-T4 interactions in the pathogenesis of PAH has been suggested since T4 inhibits MIF-induced ERK 1/2 phosphorylation in macrophages; T4 inhibits MIF activation of NF κB RelA/p65 in fibroblasts; and MIF inhibits T4-induced CXCR2 mRNA accumulation in vascular smooth muscle cells^131^. Thus, a schematic of potential mechanisms in vascular cells in hypoxia-induced altered MIF-T4 interactions is shown in Figure 2.

## Inhibition of MIF in pulmonary hypertension

Studies from our group have found significantly increased plasma MIF concentrations in individuals with primary PH or PH secondary to interstitial lung disease, compared to control subjects^45^. Therefore, we examined the effect of MIF lung fibroblast growth and showed that the increased hypoxia-induced proliferation was MIF dependent. Furthermore, in a mouse model of hypoxia-induced pulmonary hypertension, the pulmonary vascular remodeling, increased right ventricular systolic pressures and right sided cardiac hypertrophy were all significantly decreased in the presence of a small molecule inhibitor of MIF. This suggests that MIF plays a significant role the development of PH.

It should be noted, however, while in human idiopathic pulmonary arterial hypertension there is a two- to four-fold higher prevalence in postpubertal women than in men^132^, there is an opposite male-sex bias in the hypoxia, or monocrotaline rodent models of pulmonary hypertension^5,133,134^.

**Figure 3.**
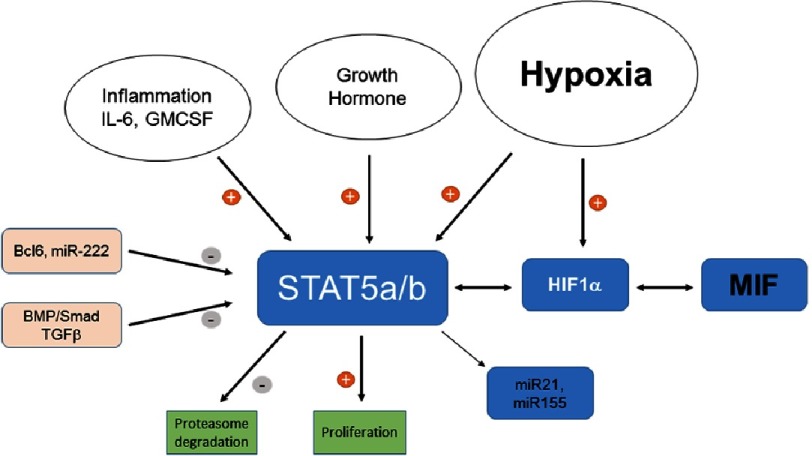
Relation between STAT5 and MIF in PH. Recent studies have highlighted the role of STAT5 in the pathogenesis of PH, especially in the sex bias associated with the pathology^135,136^. STAT5 is the regulator of multiple genes. There is a functional interdependence between MIF and HIF1 *α*, which can also impinge on STAT5 activity.

In associated studies we examined the role of the signal transducer and activator of transcription 5 (STAT 5) in the development of PH, and showed decreased STAT5 expression in the obliterative lesions of human idiopathic PAH, and that deletion of STAT5 from the vascular smooth muscle cells abrogated the male bias^135^. This is of relevance since STAT5 can act as a mediator in hypoxia-mediated gene expression^136^, and that, at least in some cells, MIF can promote intracellular signaling by STAT5^137^ (Figure 3).

In addition to the pulmonary pathology, clinical studies suggest that PAH is also associated with cognitive impairment, depression and anxiety. Recent studies have shown, in the hypoxic mouse model, that hypoxia induces increased MIF accumulation within the hippocampus (at both level of mRNA and protein), and metabolic, biochemical, and electro-physiological changes within the hippocampus were associated with cognitive dysfunction.^138,139^. These hypoxia-driven changes were corrected by the administration of an MIF inhibitor^138,140^. However, it remains unclear whether the cognitive dysfunction was corrected by direct inhibition of the increased MIF within the hippocampus, or as a result of the improvement in pulmonary function.

## MIF inhibition in congenital diaphragmatic hernia (CDH)

CDH is identified in around 1/3000 newborns^141^. It results from a defective diaphragm allowing the protrusion of abdominal tissues into the thorax, thereby interfering with normal lung development leading to lung hypoplasia and persistent pulmonary hypertension of the newborn^141^. Rodent models of the condition have been developed, and often use administration of a single dose of 2,4-dichlorophenyl-P-nitrophenyl (Nitrofen) on the tenth gestational day^142^. Poor vascular growth in the CDH rat model is associated with poor lung growth. The exact mechanism of defective angiogenesis associated with CDH is not fully understood. However, studies show that inhibiting the MIF activity in the rat CDH model results in higher expression of VEGF and Tie-2 receptor while normalizing Sflt-1. Together, these molecular changes lead to a significant improvement in pulmonary angiogenesis as well as lung development as shown by CT and histological studies^143^.

## MIF and other new pathways

In addition to being a potent vascular vasodilator, nitric oxide (NO) enhances angiogenesis by activating endothelial cell growth and tube formation^144^. Neonatal rats with CDH were treated with ISO-92 and we observed a significant increase of phosphorylated eNOS (P-eNOS), which is known to increase NO production.

This was also associated with decreases in both arginase-1 and -2 expression. Arginase is a urea cycle enzyme which competes with endothelial NO synthetase (e NOS) and inhibits NO synthesis via a common substrate, L-arginine. Hypoxia upregulates the expression of both arginase enzymes^145^.

In our study, among neonates with CDH, both arginase-1 and -2 enzymes were overexpressed significantly in comparison to healthy control neonates. This may suggest that, both NO production and its bioavailability were significantly compromised among neonates with CDH.

We have shown that treating pregnant adult rats with ISO-92 after inducing CDH around day 8-9 of gestation, significantly decreased both arginase-1 and -2 expression, which is known to eventually increase NO production and its bioavailability. Accordingly, we postulate that inhibition of both arginase-1 and -2, could be the mechanism through which inhibition of MIF activity (using ISO-92 in our model), can lead to increase NO bioavailability *in utero,* thereby improving pulmonary angiogenesis and lung development^146^.

## Conclusion and future studies

Macrophage migration inhibitory factor is a key mediator of inflammatory responses and innate immunity and has been implicated in the pathogenesis of several inflammatory and autoimmune diseases. MIF’s role in the pathogenesis of PAH, induced by chronic hypoxia, or associated with chronic lung diseases, or idiopathic, has been explored in many studies. The link between endothelial dysfunction and MIF in animals models with chronic PAH has been established. Other studies highlighted MIF role as a biomarker for the assessment of PAH associated with chronic obstructive lung diseases. Discovering the role played by T4 as a natural ligand inhibitor of MIF’s inflammatory activity opens the door for new therapeutic role of anti-MIF’s, as shown in preclinical and clinical data, which suggest that blocking the inflammatory active site of MIF may both reduce inflammatory responses and improve the availability of T4. Preclinical data using different anti-MIF’s in different animal models with chronic and severe forms of pulmonary hypertension are very promising. Whether inhibition of MIF or its oxidized forms may offer promising therapy in PAH, needs to be elaborated in future human interventional studies.
